# Development of Antioxidant Composites Based on Reprocessed PLA Reinforced with Bacterial Cellulose Obtained from Kombucha Fermentation in an Infusion of Cauliflower Stems

**DOI:** 10.3390/polym18192390

**Published:** 2026-09-30

**Authors:** Daniela A. Cerro, Pedro Pacios, Ángel Agüero Rodríguez, Eva Hernández-García, Marina P. Arrieta

**Affiliations:** 1 Departamento de Ingeniería Química Industrial y del Medio Ambiente, Escuela Técnica Superior de Ingenieros Industriales, Universidad Politécnica de Madrid (ETSII-UPM), 28006 Madrid, Spain; daniela.cerro@externos.upm.es (D.A.C.); p.pacios@alumnos.upm.es (P.P.); angel.agrodriguez@externos.upm.es (Á.A.R.); 2 Departmental Section of Galenic Pharmacy and Food Technology, Veterinary Faculty, Complutense University of Madrid, Av. Puerta de Hierro, s/n, 28040 Madrid, Spain; evaher09@ucm.es; 3 Grupo de Investigación, Polímeros, Caracterización y Aplicaciones (POLCA), 28006 Madrid, Spain; 4 Centro de Investigación en Materiales Estructurales (CIME), Universidad Politécnica de Madrid, C/Profesor Aranguren 3, 28040 Madrid, Spain

**Keywords:** bacterial cellulose, kombucha, cauliflower stem, reprocessed poly(lactic acid) (rPLA), biodegradable composites, antioxidant materials, compostability

## Abstract

In this study, cauliflower stems were used as an infusion medium for kombucha fermentation to produce kombucha bacterial cellulose (KBC). The obtained KBC was incorporated into reprocessed poly(lactic acid) (rPLA) films as a reinforcing filler. Cauliflower stem powder (CS) was also evaluated for comparison. Films containing 1 and 3 wt.% of each filler were characterized in terms of their mechanical, thermal, physicochemical, and morphological properties, surface wettability, water vapor barrier properties, antioxidant activity using a fatty food simulant, and disintegration under laboratory-scale composting conditions. The results showed that both fillers reduced the mechanical strength of the films without significantly affecting their thermal transitions, chemical structure, or surface wettability. rPLA-KBC-reinforced films exhibited the best overall performance for the intended use, with lower reductions in mechanical and barrier properties and providing antioxidant activity to rPLA-based systems, reaching radical scavenging activities of approximately 15% and 26% for films containing 1 and 3 wt.% of KBC, respectively. In addition, all formulations completely disintegrated under composting conditions within approximately 21 days. These findings demonstrate the potential of combining agricultural and industrial waste to develop sustainable antioxidant rPLA-based materials for food packaging applications.

## 1. Introduction

Sustainability and efficient waste management have become imperative, driving a critical transition from a linear economy, based on the “produce, use, and dispose” model, toward a circular economy focused on the efficient and systematic valorization of resources. This paradigm shift aims to close product life cycles, transforming traditional waste into high-value secondary raw materials. In this regard, the recovery of plant-based byproducts has emerged as one of the most promising strategies, as it not only mitigates environmental impact but also allows for the exploitation of their complex chemical composition for the development of new functional materials [1,2].

Under this premise, the generation of agro-industrial waste has shifted from being viewed solely as a significant environmental challenge to being recognized as an underutilized technical opportunity. A prominent example is cauliflower (*Brassica oleracea*), whose cultivation and industrial processing generate substantial amounts of residual biomass, with discarded outer leaves and stems accounting for approximately 30–50% of the total biomass, depending on the production chain [3]. Despite their high content of valuable phytochemicals, these by-products are still largely underutilized and are commonly discarded or destined for low-value applications [3]. This residual biomass possesses significant interest due to the bioactive potential due to its richness in antioxidant compounds that can be successfully exploited as polymer additive [4]. However, despite its promising composition, its application as a functional reinforcement in biodegradable polymeric materials remains scarcely explored. Harnessing this potential requires the development of innovative valorization strategies capable of effectively integrating these agro-industrial residues into bio-based value chains [2].

Compared to conventional polyesters such as polyethylene terephthalate (PET), polylactic acid (PLA) is relatively new but increasingly used material, and represents a leading bioplastic alternative to fossil-based polymers in the food packaging field due to its good balance between processability, overall properties, inherent compostability, and competitive price [5]. Although biobased, PLA-based packaging uses large quantities of primary raw materials, while recycling rates in single-use industrial applications still remain low [6]. In this sense, although PLA can be technically separated from other plastics in the waste stream using established sorting technologies such as near-infrared (NIR) spectroscopy, complemented by more innovative approaches such as hyperspectral imaging [7,8], currently, PLA recycling remains very limited because of the relatively low volumes of PLA reaching post-consumer waste streams. In fact, NIR-based sorting technologies have demonstrated that PLA with high purity (91–95%) can be obtained with sorting yields of 73–78% [9]. However, dedicated PLA sorting is estimated to become economically feasible when PLA represents approximately 1–5% of the total packaging waste streams [9].

The current European regulation aims to lower the use of virgin polymeric materials, while safely increasing the use of recycled plastics in the packaging field [10]. However, for food safety concerns, the current legislation does not allow the direct use of plastics from conventional recycling streams for food contact materials (FCM) [10,11], as the presence and potential migration of intentionally and non-intentionally added substances (NIAS) into foodstuff can potentially affect both food quality and consumer safety [10,12].

Although PLA is compostable, composting as solely end-of-life option may be limited by existing infrastructure, whereas mechanical recycling currently represents a viable route that allows the material value to be retained within the production cycle reducing the consumption of virgin resources showing less environmental impact compared to chemical recycling and composting [5,13]. Meanwhile, mechanically recycled PLA can be further composted [14,15]. Recently, Silva et al. [16] simulated postconsumer PLA by exposing PLA to food simulants and evaluated its subsequent mechanical recycling process. A reduction in molecular weight was observed due to service and subsequent secondary melt-extrusion cycles. Nevertheless, it did not significantly affect molecular weight reduction due to service. Further, we observed secondary melt-extrusion cycle, but it did not have a significant impact on the mechanical performance of PLA [16]. However, postconsumer PLA may be exposed to contaminants during service and disposal, which may hinder its subsequent recycling for FCM. In contrast, PLA industrial scaling entails the generation of significant scraps and burrs that requires efficient management, with reprocessing (rPLA) standing as an effective strategy to mitigate these industrial residues [12,15]. In this regard, in previous work NIAS and metals were assayed, and it was observed that substances with specific migration limits remained below applicable thresholds suggesting that rPLA represents a viable option for FCM development [12]. This process is an imperative step toward consolidating the circular economy, allowing such material parts to be reintegrated into the production cycle [9]. Nevertheless, subjecting PLA to repeated thermal cycles triggers thermo-mechanical degradation that can compromise its structural quality, limiting its applications [17,18,19].

In addition, PLA presents intrinsic limitations for food packaging applications, including limited thermal stability, mechanical and barrier properties, as well as high water sensitivity [20,21,22], which can be further marked in mechanically recycled PLA [12,17,19]. These drawbacks can be mitigated through the incorporation of suitable reinforcing fillers capable of improving mechanical performance and creating a more tortuous pathway for water vapor diffusion within the polymer matrix [17,23]. Such an approach enables the development of functional biocomposites while maintaining their inherent disintegration character at the end of their useful life [24].

Among others, natural cellulose derivatives have been widely used as PLA reinforcement due to their cellulosic hierarchical structure, providing improved mechanical and barrier properties to PLA-based materials, thus allowing us to obtain fully biobased and compostable food packaging. However, while the traditional extraction of cellulose from plants involves the previous elimination of lignin and hemicellulose, the bacterial cellulose production leads directly to pure cellulose [22,25,26]. One interesting source of bacterial cellulose is that produced from kombucha beverage, as kombucha cultures gradually lose their fermentative activity after repeated fermentations and SCOBYs. Their valorization as a renewable cellulose source from the HoReCa (HOtel, REstaurant and CAtering) sector represents a sustainable strategy to produce pure cellulose while reducing waste [22].

Therefore, an innovative biotechnological route to addressing the limitations of rPLA involves the use of kombucha symbiotic cultures of bacteria and yeast (SCOBY) [17]. This process enables the valorization of agro-industrial byproducts as fermentation substrates as well as kombucha SCOBY, transforming them into high-purity functional reinforcements [17,27,28,29]. Consequently, kombucha fermentation has emerged as a versatile biotechnological platform to produce not only a probiotic beverage, but also sustainable functional materials from the production waste. Recent research has demonstrated that various plant infusions and agro-industrial residues, such as spent coffee grounds or yerba mate waste, can be successfully utilized to produce bacterial cellulose (BC) with properties suitable for incorporation into biodegradable polymer matrices [22].

The relevance of this approach stems from the dual functionality of bacterial cellulose (BC) as its dense nanofibrillar network acts as reinforcing agent, improving the mechanical and thermal performance of polymer composites [28,29], while also serving as a carrier of bioactive compounds retained during fermentation, enabling the development of active packaging materials with antioxidant properties [22,30]. This multifunctionality is highly dependent on both the fermentation conditions and the nature of the plant substrate employed for the infusion media for the fermentation [31,32]. Therefore, selecting an appropriate agricultural substrate is essential to maximize both the reinforcing effect and the antioxidant functional performance of the resulting materials.

There is a compelling need to compare cauliflower stem powder (CS) with kombucha-derived bacterial cellulose fermented in CS (KBC). While the use of powder reduces processing costs and acts as a conventional filler, bacterial cellulose offers superior functional performance owing to its nanometric structure and high crystallinity, which promotes stronger interfacial adhesion with the polymer matrix and generates a more effective tortuous barrier against moisture than conventional lignocellulosic fibers [33,34]. To the best of our knowledge, there were no previous studies systematically comparing these two valorization strategies using cauliflower powder—which is an agricultural residu—as active additive for reprocessed PLA films, either directly used as filler or as an infusion powder for kombucha beverage production.

In the framework of this circular economy approach, the present work proposes a novel strategy based on the production of kombucha bacterial cellulose (KBC) from cauliflower stems, an underutilized agricultural byproduct, for its application as a reinforcing filler for reprocessed PLA (rPLA) matrix, addressing industrial valorization of PLA obtained from production scrap, and thus representing a realistic post-industrial recycling scenario. To this end, the influence of CS and KBC loadings (1 and 3 wt.%) on the thermal, mechanical, physicochemical, morphological, properties of compression-molded rPLA films were evaluated. Additionally, functional properties of the developed materials as surface wettability, water barrier and the antioxidant activity from the released bioactive compounds into a fatty food simulant were also investigated. Finally, disintegrability under composting conditions was assessed at a laboratory scale to obtain insights into the disintegration behavior of such materials. This was performed to propose compostability as an end-of-life option. Therefore, this study provides insights into the potential of cauliflower stem powder, as a reinforcing filler and active compound, for the production of kombucha-derived bacterial cellulose as a sustainable reinforcement used in the development of functional rPLA-based materials for active food-packaging applications.

## 2. Materials and Methods

### 2.1. Materials

Polylactic acid (PLA) Ingeo™ Biopolymer 2003D (NatureWorks^®^, Minnetonka, MN, USA) was used as the polymer matrix. The material presented a melt flow index (MFI) of 6.5 g/10 min, determined at 190 °C under a 2.16 kg load using a Metrotec MFI-100 analyzer (Techlab Systems, Lezo, Spain), as previously reported by Sepúlveda-Carter et al. [12]. For bacterial cellulose production, a symbiotic culture of bacteria and yeast (SCOBY), consisting of a stock culture kombucha (SCK), was used as the starter culture [22]. Cauliflower stems obtained from a local greengrocery were used to prepare the cauliflower stem infusion for kombucha fermentation and the cauliflower stem powder (CS). Commercial sucrose (Azucarera Iberia SLU, Madrid, Spain) was used as the carbon source during fermentation. Ethanol (99.8% purity) and 2,2-diphenyl-1-picrylhydrazyl (DPPH) were purchased from Sigma-Aldrich S.A. (Madrid, Spain) and used for the antioxidant activity determination.

### 2.2. Materials Production and Processing

#### 2.2.1. Production of Kombucha Bacterial Cellulose (KBC)

A SCOBY was produced in the laboratory from a previously prepared stock culture kombucha of spent coffee ground kombucha (SCK), following the procedure described by Agüero et al. [22]. The obtained SCOBY was transferred into a glass vessel containing 1 L of SCK and 2 L of cauliflower stem infusion, prepared according to [22]. The cauliflower stem infusion was prepared using 15 g of cauliflower stems previously dried in a J.P. Selecta oven (Barcelona, Spain) at 50 °C for 72 h. The dried stems were added to 1 L of boiling water and infused at 80 °C for 15 min. After filtration, the infusion was mixed with an additional 1 L of water containing 300 g of dissolved sucrose, resulting in final concentrations of 7 g/L of cauliflower solids and 150 g/L of sucrose. The glass vessel was covered with paper to allow the diffusion of CO_2_ during fermentation and incubated at 25 ± 2 °C and 50% RH for 30 days. At the end of the fermentation, several layers of newly produced SCOBY disks were formed on the liquid surface. The obtained SCOBY disks were recovered, rinsed three times with deionized water, and sterilized in a steam autoclave at 121 °C and 100 kPa for 15 min (JP Selecta, Barcelona, Spain). The sterilized SCOBY disks were cut into small pieces and mixed with deionized water at a 1:3 (*w*/*v*) ratio. The resulting suspension was homogenized using an Ultra-Turrax 45/2 (IKA-Werke, Germany) in four cycles of 30 s at 30,000 rpm, followed by sonication using a Q125 Sonicator (Fisher Scientific, Waltham, MA, USA) equipped with a CL-18 probe at 80 W for three cycles of 5 min. The sonicated suspension was vacuum-filtered through a Büchner funnel, dried in an oven at 60 °C for 48 h, and milled into a fine powder using a cutter grinder (IKA-Werke, Staufen, Germany). The obtained powder was then sieved to a particle size below 50 µm and is hereafter referred to as kombucha bacterial cellulose (KBC).

#### 2.2.2. Preparation of Reprocessed PLA (rPLA) and rPLA-Based Composites

rPLA was prepared as described in previous works to simulate the valorization of post-industrial PLA waste (e.g., defective parts, edges, burrs, and production scraps generated during PLA manufacturing) [12,15]. Briefly, virgin PLA pellets were melt-extruded to produce neat PLA filament in a 3Devo Composer 350 extruder (3Devo, Utrecht, The Netherlands) with a temperature profile of 185/190/190/185 °C (from die to hopper) and a screw speed of 5 rpm. The obtained PLA filaments were pelletized and subsequently re-extruded under the same processing conditions to obtain reprocessed PLA (rPLA). The resulting rPLA exhibited a melt flow index (MFI) of 9.2 g/10 min [12].

The rPLA was subsequently incorporated with 1 or 3 wt.% of cauliflower stem powder (CS) or kombucha bacterial cellulose (KBC) to produce the corresponding rPLA-based composites. The selected filler loadings of 1 and 3 wt.% were based on our previous studies with kombucha cellulose particles derived from HoReCa waste [17,22], where higher KBC contents (5 wt.%) resulted in an increased particle agglomeration and morphological heterogeneities [21], while lower filler contents (1–3 wt.%) showed reduced agglomeration and provided additional functionalities, such as antioxidant activity [17,22].

The composite systems were prepared following the procedure previously reported by González-Serrud et al. [15] for the incorporation of lignocellulosic fillers into rPLA matrices. The previously dried rPLA pellets (60 °C for 8 h) were mixed with 1 and 3 wt.% of CS or KBC in a sealed glass jar to promote filler distribution before the melt-blending extrusion step. All formulations were subsequently extruded using the same temperature profile (185/190/190/185 °C, from die to hopper) at a screw speed of 5 rpm in a 3Devo Composer 350 extruder (3Devo, Utrecht, The Netherlands)). All extruded filaments were pelletized using a Felfil Plastic Shredder+ Model 500 (Felfil, Turin, Italy) and dried at 40 °C for 48 h in a vacuum oven (J.P. Selecta, Barcelona, Spain), prior to film production.

#### 2.2.3. Film Production by Compression Molding

Approximately 1.5 g of each composite pellets were placed between the heated plates of a Mr. Hide WTRP-10T Rosin press (Tarragona, Spain) and processed by compression molding to obtain the film formulations (Table 1). The processing conditions were previously optimized to obtain homogeneous films with complete polymer melting while minimizing the presence of trapped air bubbles following a previous reported procedure [21]. The press was set at 200 °C, corresponding to an actual sample temperature of approximately 160 °C due to heat losses between the heated plates. Compression was carried out using an increasing pressure cycle: (i) 2 min without pressure (preheating), (ii) 3 MPa for 1 min, (iii) 5 MPa for 2 min, and (iv) 30 MPa for 5 min. After compression, the films were allowed to cool at room temperature for approximately 10 min before being removed from the press. The composition and formulation codes of all materials are summarized in Table 1.

### 2.3. Characterization Techniques

#### 2.3.1. Viscosity-Average Molecular Weight

The intrinsic viscosity of the PLA and rPLA pellets and films was determined using chloroform (CHCl_3_) as a solvent with a type 1C Ubbelohde viscometer in Selecta VB-1423 water bath (J.P. Selecta, Abrera, Spain) at 25 °C. For that, each PLA-based sample was dissolved in CHCl_3_ and at least four concentrations between 0.00135 and 0.00619 g/mL were assessed. The reduced and inherent viscosities were calculated and plotted as a function of polymer concentration according to the Huggins and Kraemer approaches, respectively and then the intrinsic viscosity [η] was obtained as the average of the intercepts of both linear extrapolations at zero concentration. The dispersion between both intercepts was considered as the uncertainty associated with [η]. The intrinsic viscosity [η] was further used to estimate the viscosity-average molecular weight (M_v_) using the Mark–Houwink–Sakurada equation (Equation (1)) and the uncertainty associated with [η] was propagated to M_v_.

(1) $$\left[\eta \right]=K\times M_{v}^{a}$$

in which *K* and *a* for the PLA polymer are 1.53 × 10^−2^ and 0.759, respectively [12].

#### 2.3.2. Fourier Transform Infrared (FTIR) Spectroscopy

Fourier transform infrared (FTIR) spectra of films were recorded using a Jasco FT/IR-4X spectrometer (Jasco, Easton, MD, USA) equipped with an attenuated total reflectance (ATR) accessory. Spectra were acquired using Spectra Manager 2005 software by averaging 64 scans over the 4000–400 cm^−1^ spectral range with a resolution of 4 cm^−1^ at room temperature.

#### 2.3.3. Thermogravimetric Analysis

Dynamic thermogravimetric analysis (TGA) was performed in duplicate using a SETARAM thermogravimetric analyzer (SETARAM, Caluire, France). Approximately 5 mg of each sample was placed in an open platinum crucible and heated from 40 to 800 °C at a heating rate of 10 °C min^−1^ under a nitrogen atmosphere with a flow rate of 30 mL min^−1^. The onset temperature was determined at 5% weight loss (T_5_%) from the TGA curves, while the temperature of maximum degradation rate (T_max_) was obtained from the derivative thermogravimetric (DTG) curves.

#### 2.3.4. Differential Scanning Calorimetry (DSC)

Differential scanning calorimetry (DSC) was performed using a SETLINE DSC (SETARAM, Caluire, France) under a nitrogen atmosphere. Approximately 5–7 mg of each sample was analyzed. The first heating scan was carried out from 25 to 200 °C at a heating rate of 10 °C min^−1^ to erase the thermal history. The samples were then cooled to 25 °C at the same rate, followed by a second heating scan from 25 to 250 °C at 10 °C min^−1^. All analyses were performed in duplicate. The degree of crystallinity (χ_c_) was calculated from the second heating scan according to Equation (2).

(2) $$\chi_{c}=\frac{\;{\Delta H}_{m}-{\Delta H}_{\mathrm{cc}}}{{\Delta H}_{m}^{{}^{\circ}}}\cdot \frac{1}{W_{\mathrm{PLA}}}100$$

where Δ*H*_m_ is the melting enthalpy, Δ*H*_cc_ is the cold crystallization enthalpy, ${\Delta H}_{m}^{{}^{\circ}}$ is the melting enthalpy of 100% crystalline PLA (93 J g^−1^) [20], and WPLA is the weight fraction of PLA in each formulation.

#### 2.3.5. Field Emission Scanning Electron Microscopy (FESEM)

The cross-sectional morphology of the films was examined by field emission scanning electron microscopy (FESEM) using a JSM-IT700HR scanning electron microscope (JEOL Ltd., Tokyo, Japan). Film specimens were cryo-fractured in liquid nitrogen and mounted on aluminum stubs using conductive carbon tape. Prior to observation, the samples were sputter-coated with a thin carbon layer to improve electrical conductivity. Micrographs were acquired under high vacuum at magnifications of 500× and 10,000×. The filler-related features were measured by means of ImageJ software (version 1.51k, Java 1.6.0_24, Wayen Rasband, US National Institutes of Health, Bethesda, MD, USA).

#### 2.3.6. Mechanical Properties

The tensile properties of the films were evaluated using a Shimadzu AGS-X universal testing machine (Shimadzu Corporation, Kyoto, Japan) equipped with a 100 N load cell and operated using TRAPEZIUM X software (v1.5.4). Tensile tests were performed at a crosshead speed of 10 mm min^−1^ with an initial grip separation of 30 mm, following ISO 527-1:2020 [35]. Test specimens were prepared using a pneumatic die cutter (Instruments J. Bot S.A., Cabrils, Spain) with dimensions of 20 mm × 2 mm. The average thickness measured for each formulation was used for the stress calculations. The Young’s modulus (E), tensile strength at break (σ), and elongation at break (ε) were determined from the stress–strain curves. The reported values correspond to the average of at least five specimens per formulation.

#### 2.3.7. Water Vapor Transmission Rate (WVTR) Tests

The water vapor transmission rate (WVTR) of the films was determined using the gravimetric cup method. Film specimens were mounted in permeability cups (TQC Sheen, Capelle aan den IJssel, The Netherlands) containing approximately 2 g of dry silica gel as a desiccant. The exposed film area was 10 cm^2^. The cups were placed in a desiccator containing a saturated KNO_3_ solution to maintain a relative humidity of approximately 90% at 21.4 ± 0.5 °C. The cups were weighed every hour for 7 h and again after 24 h. The WVTR (g day^−1^ cm^−2^) was calculated according to Equation (3).

(3) $$\mathrm{WVTR}=\frac{240\times \left(m_{t}-m_{0}\right)}{A\times t}$$

where m_t_ is the mass (mg) of the cup at time t (h), m_0_ (mg) is the initial mass of the cup and A (cm^2^) is the exposed area of the films. The WVTR values were normalized to 100 µm.

#### 2.3.8. Static Water Contact Angle Measurements (WCA)

The surface wettability of the films was evaluated by static water contact angle (WCA) measurements using a standard goniometer (Ossila BV, Leiden, The Netherlands) equipped with a high-resolution camera and Ossila software (v4.1.4) for contact angle analysis. Measurements were performed at room temperature by depositing five 10 μL droplets of distilled water on randomly selected regions of each film surface. Water contact angles were recorded 30 s after droplet deposition. Five independent droplets (*n* = 5) were analyzed for each formulation, and the WCA values are reported as the mean ± standard deviation.

#### 2.3.9. Specific Migration Test of Antioxidant Activity

rPLA-based films were immersed in 9 mL of food simulant D1 (50% *v*/*v* ethanol–water) in glass vials. Two film specimens were placed in each vial, (area-to-volume ratio = 6 dm^2^/L) and migration tests were performed in triplicate under immersion conditions at 40 °C for 10 days. After incubation, the films were removed and the volume of the food simulant was verified to ensure the absence of solvent loss due to evaporation. The antioxidant activity of the compounds released into the food simulant was determined using the DPPH radical scavenging assay. Briefly, 900 μL of food simulant was mixed with 100 μL of a 0.002 M DPPH solution prepared in ethanol. The mixtures were incubated in the dark for 15 min, and the absorbance was measured at 517 nm using a Shimadzu 1900i UV–Vis spectrophotometer (Shimadzu Corporation, Kyoto, Japan). The antioxidant activity was calculated by the determination of the radical scavenging activity (RSA) according to Equation (4).

(4) $$\mathrm{RSA}\;\left(\%\right)=\frac{A_{c}-A_{i}}{\mathrm{Ac}}\times 100$$

#### 2.3.10. Disintegration Tests Under Composting Conditions

The disintegration of the films under composting conditions was evaluated at a laboratory scale according to ISO 20200:2024 [36]. Film specimens (15 mm × 15 mm) were cut, and seven specimens of each formulation were placed in textile mesh bags to facilitate handling, recovery, and exposure to the composting medium [21]. Before composting their initial dry mass (m_i_) was determined using an AX125D semi-micro analytical balance (OHAUS Europe GmbH, Nänikon, Switzerland). The mesh bags were buried at a depth of 4–6 cm in perforated plastic containers acting as reactors and containing a synthetic compost. The compost was prepared using the following dry-weight composition: 40% sawdust, 30% rabbit feed, 10% mature compost, 10% corn starch, 5% sugar, 4% corn seed oil, and 1% urea, with a final moisture content of approximately 50% (*w*/*w*). The reactors were incubated in a ventilated oven under aerobic conditions at 58 °C. Aerobic conditions were maintained by periodically mixing the composting medium, while moisture was restored by adding water when necessary. Film specimens were recovered after 1, 4, 7, 11, 14, and 21 days of composting test. Adhered compost residues in disintegrated films were removed by washing the recovered samples with distilled water. The recovered disintegrated film samples were dried under vacuum at 40 °C for 24 h and weighed to determine the residual dry mass (m_r_). The compost and recovered film specimens were photographically documented. The degree of disintegration (D, %) was calculated according to Equation (5):

(5) $$D\;\left(\%\right)=\;\frac{m_{i}-\;m_{r}}{m_{i}}$$

#### 2.3.11. Statistical Analysis

Statistical analyses were performed for the experimental data, when applicable, using GraphPad Prism (GraphPad Software, version 8.0, Boston, MA, USA). Data are presented as mean ± standard deviation. Statistical differences among formulations were evaluated by one-way analysis of variance (ANOVA) followed by Tukey’s multiple comparison test. For datasets that did not satisfy the assumptions of ANOVA, Welch’s ANOVA followed by Dunnett’s T3 multiple comparison test was applied. Differences were considered statistically significant at *p* < 0.05.

## 3. Results and Discussion

### 3.1. Production of Kombucha Bacterial Cellulose (KBC)

The fermentation process of kombucha bacterial cellulose (KBC) in a cauliflower stem was successfully achieved, resulting in a slight increase in the mass of the original inoculum disk (from approximately 48 g to 54 g) and the formation of new cellulose disks, reaching a total wet biomass of around 388 g after 30 days. Considering both the growth of the original cellulosic disk and the newly formed biomass, this corresponded to an overall wet biomass increase of about 812% (*w*/*w*) relative to the initial wet inoculum and a net wet biomass production of around 197 g L^−1^ of wet bacterial cellulose after 30 days of cultivation. It should be noted that this value represents hydrated biomass rather than dry bacterial cellulose yield [17,22,37]. In this regard, Chaussé et al. reported higher bacterial cellulose yields under optimized laboratory conditions using specifically isolated strains, such as *Komagataeibacter europaeus*, in a defined medium containing glucose, peptone, yeast extract, sodium acetate, and ethanol, rather than in a conventional tea-based kombucha infusion, reaching kombucha bacterial cellulose yields of 496 ± 14 g L^−1^ on a hydrated basis after 10 days of cultivation but only 19 ± 1 g L^−1^ on a dry basis, highlighting the strong influence of the high water content of bacterial cellulose on production values [38]. Therefore, direct comparison between wet biomass production and dry bacterial cellulose yields are not appropriate, while comparisons based on wet biomass should also be interpreted with caution because cultivation conditions, fermentation time, inoculum characteristics, and yield calculations differ substantially among studies. Nevertheless, the wet biomass production observed in the present work demonstrates that cauliflower stem infusion can effectively support bacterial cellulose production. Unlike conventional systems based on defined synthetic media such as Hestrin–Schramm medium, which relies on refined carbon and nitrogen sources, the proposed approach employed cauliflower stem residues as a low-cost source of nitrogen and micronutrients, requiring supplementation only with sucrose as the carbon source. Thus, these findings highlight the potential of cauliflower stem residues as an alternative fermentation substrate, supporting their valorization within a circular economy approach while providing a sustainable route for producing bacterial cellulose with the additional advantages of being rich in antioxidant compounds [4]. However, determination of dry KBC productivity and carbon utilization efficiency would be required to quantitatively assess production efficiency and sustainability for the upscaling of the process.

### 3.2. Physicochemical Characterization of the Films

The intrinsic viscosity was evaluated to assess the effect of processing (melt extrusion and film forming) and reprocessing by melt extrusion on the polymer molecular structure and further related to the viscosity-average molecular weight (M_v_) of PLA and rPLA pellet and films (Table 2). The M_v_ of PLA decreased by 7.8% after reprocessing, suggesting that the mechanical recycling process induced some polymer chain degradation. The reduction on the M_v_ values of PLA was comparable to those previously reported for mechanically recycled PLA processed by melt extrusion [12,14,15,16]. A further decrease was observed after film processing for PLA and rPLA film, indicating a progressive reduction in molecular weight associated with the thermo-mechanical degradation of PLA during processing.

**Figure 1:**
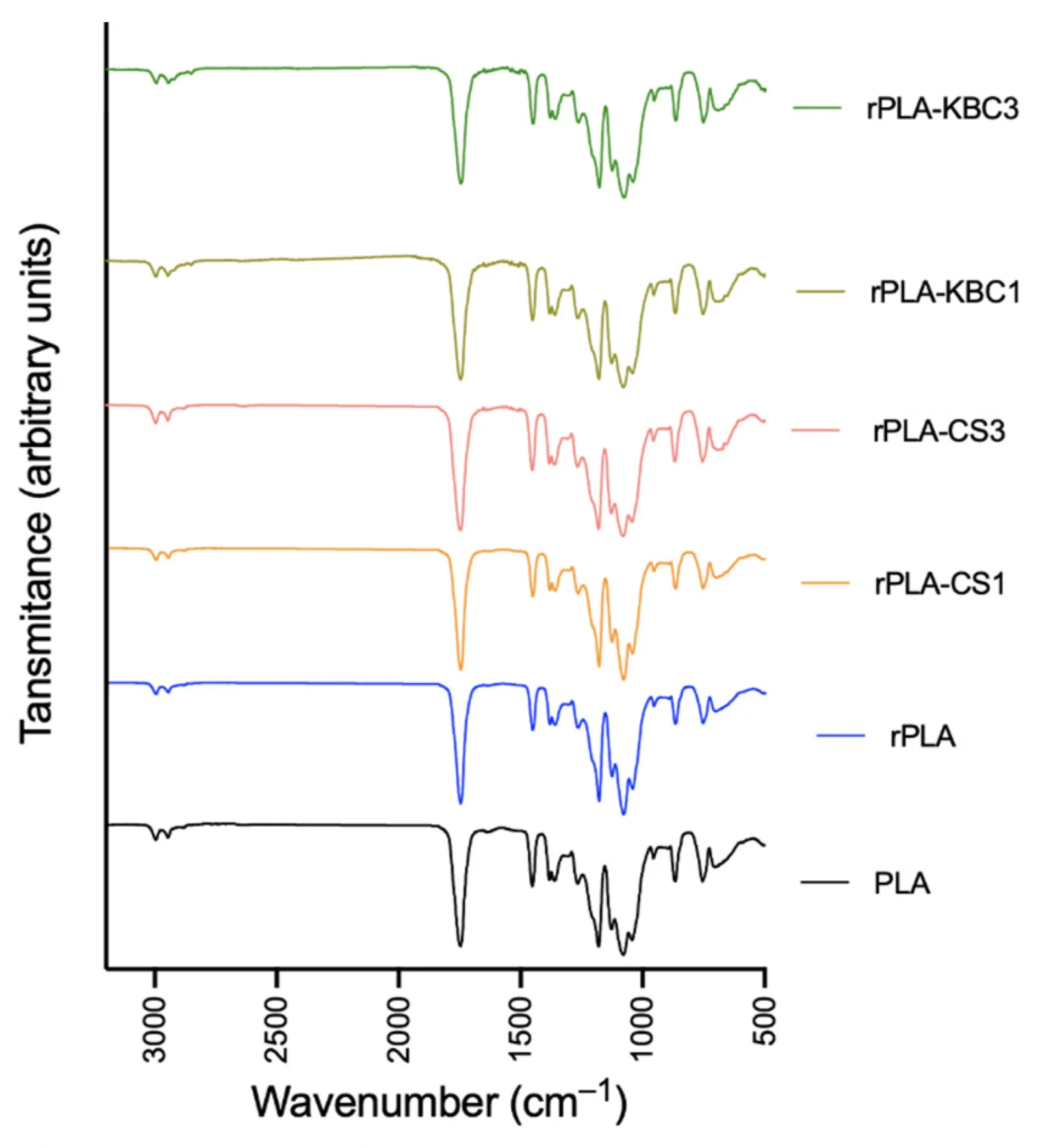
FTIR spectra of PLA-based films reinforced with cauliflower stem powder (CS) and kombucha bacterial cellulose (KBC).

ATR-FTIR spectroscopy was used to evaluate the effect of reprocessing and CS and KBC filler incorporation on the physicochemical structure of the rPLA matrix. As shown in Figure 1, all formulations exhibited the characteristic ATR-FTIR spectrum of PLA, indicating that the polymer backbone remained largely unaffected during processing. The spectra displayed the typical absorption bands of PLA, including the asymmetric and symmetric C–H stretching vibrations at approximately 2995–2946 cm^−1^, the intense ester carbonyl (C=O) stretching band at 1747 cm^−1^, and the characteristic C–O–C stretching region between 1180 and 1040 cm^−1^ [5]. Comparison between neat PLA and reprocessed PLA (rPLA) revealed no additional absorption bands or significant spectral shifts after reprocessing, indicating that the characteristic chemical structure of PLA was preserved. Although no new bands or relevant shifts were observed in the ATR-FTIR spectra, the decrease in viscosity-average molecular weight (M_v_) after reprocessing and film processing suggests polymer chain degradation due to thermo-mechanical processing, which was not clearly detected by FTIR. Minor variations in band intensity were observed within the fingerprint region. However, these changes were insufficient to attribute specific modifications in the crystalline or amorphous domains based solely on ATR-FTIR analysis. Similar observations have been reported for rPLA-based systems, where slight variations in ATR-FTIR bands intensity were detected after reprocessing without significant changes in the characteristic spectral profile or chemical structure of PLA [12].

Likewise, the incorporation of CS and KBC did not induce new absorption bands or relevant peak shifts relative to rPLA. These findings indicate that filler incorporation did not induce detectable chemical modifications in the PLA polymer backbone under the processing conditions employed, suggesting that the interactions between rPLA and both fillers were predominantly physical rather than chemical [22].

### 3.3. Thermal Properties by TGA

**Figure 2:**
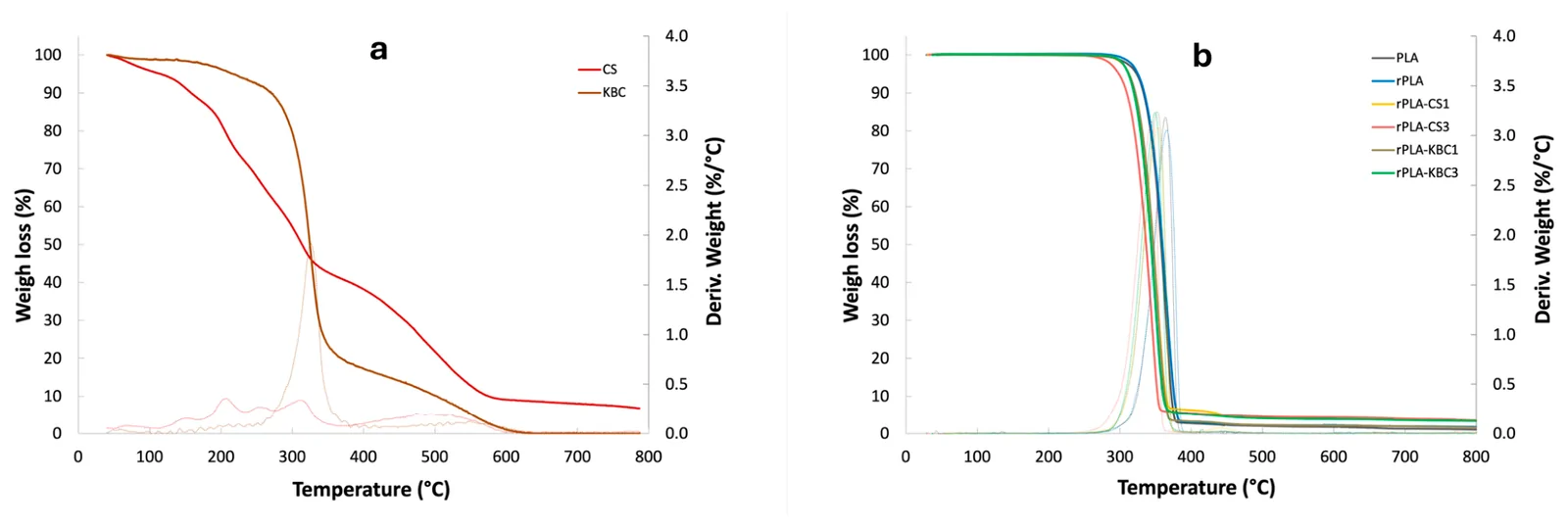
TGA and derivative thermogravimetric (DTG) curves of (**a**) cauliflower stem powder (CS) and kombucha bacterial cellulose (KBC) and (**b**) rPLA-based films reinforced with cauliflower stem powder (CS) and kombucha bacterial cellulose (KBC).

The thermal degradation behavior of the biobased fillers and the developed films was evaluated by thermogravimetric analysis (TGA), and the corresponding TGA and DTG curves are presented in Figure 2. Cauliflower stem powder (CS) exhibited a multistep degradation profile characteristic of lignocellulosic materials (Figure 2a). An initial mass loss below 100 °C was associated with moisture evaporation, followed by multiple degradation events between approximately 200 and 300 °C, mainly attributed to the decomposition of hemicellulose and other thermally less stable plant constituents [39]. The main degradation peak occurred at 207.1 °C, while the relatively high residual mass (6.7%) reflected the heterogeneous composition of the plant-derived material. In contrast, kombucha bacterial cellulose (KBC) showed a more homogeneous degradation profile (Figure 2a), characterized by a single and narrower DTG peak, with a degradation onset at 206.6 °C and a T_max_ of 327.3 °C. The lower residual mass (1.9%) further supports the reduced content of thermally stable non-cellulosic components. Similar behavior has been reported for kombucha-derived bacterial cellulose produced from coffee residues, where the simpler degradation profile was attributed to the higher compositional uniformity of bacterial cellulose compared with lignocellulosic plant materials [22].

Regarding the films, neat PLA and reprocessed PLA (rPLA) exhibited comparable thermal degradation behavior (Figure 2b), with very similar T_5%_ and T_max_ values (Table 3). These results indicate that the additional extrusion cycle did not produce noticeable changes in the thermal stability of the PLA matrix, despite the reduction in viscosity-average molecular weight observed after reprocessing. This suggests that the molecular weight reduction associated with thermo-mechanical degradation was not enough to significantly affect the thermal performance of PLA, in agreement with previous findings for mechanically reprocessed PLA [12]. The incorporation of bio-based fillers modified the thermal degradation profile of the films (Figure 2b), leading to a reduction in both the onset degradation temperature (T_5%_) and the maximum degradation temperature (T_max_) compared with PLA and rPLA. This behavior is commonly reported for PLA-based composites reinforced with lignocellulosic materials and is generally attributed to the lower intrinsic thermal stability of cellulose-based fillers, whose degradation begins at lower temperatures than the PLA matrix. The degradation products released from these fillers may also promote earlier thermal decomposition of the surrounding PLA. Among the reinforced formulations, the effect was more pronounced for the CS-containing films, particularly at 3 wt.%, as rPLA-CS3 exhibited the lowest T_5%_ and T_max_ values. This behavior is likely associated with the presence of hemicellulose and lignin in rPLA-CS-based films. In contrast, although KBC-containing films also exhibited lower degradation temperatures than neat PLA and rPLA, this reduction was less pronounced than that observed for rPLA-CS3. The higher purity of KBC than CS, together with the absence of hemicellulose and lignin fractions present in CS, may have contributed to moderating the effect of filler incorporation on thermal degradation. It is known that bacterial cellulose exhibits high affinity for phenolic compounds through non-covalent interactions such as hydrogen bonding [36]. Thus, the cellulose-rich structure of KBC may favor the retention of antioxidant compounds from the cauliflower infusion during fermentation, which could potentially contribute to limiting thermal degradation of the polymer matrix. Similar trends have been reported for PLA composites reinforced with bacterial cellulose by Agüero et al., where the degradation behavior is mainly governed by the intrinsic thermal characteristics of the lignocellulosic phase rather than by changes in the chemical structure of PLA [22,40]. The incorporation of CS and KBC also increased the residual mass after thermal degradation, particularly at the highest filler loading, which is consistent with the char-forming tendency of lignocellulosic materials. Although both fillers reduced T_5%_ and T_max_, all formulations remained thermally stable at temperatures well above those typically employed for PLA melt processing. Therefore, these variations are not expected to compromise either the processing or the intended packaging applications of the developed composites.

### 3.4. Thermal Properties by DSC

**Figure 3:**
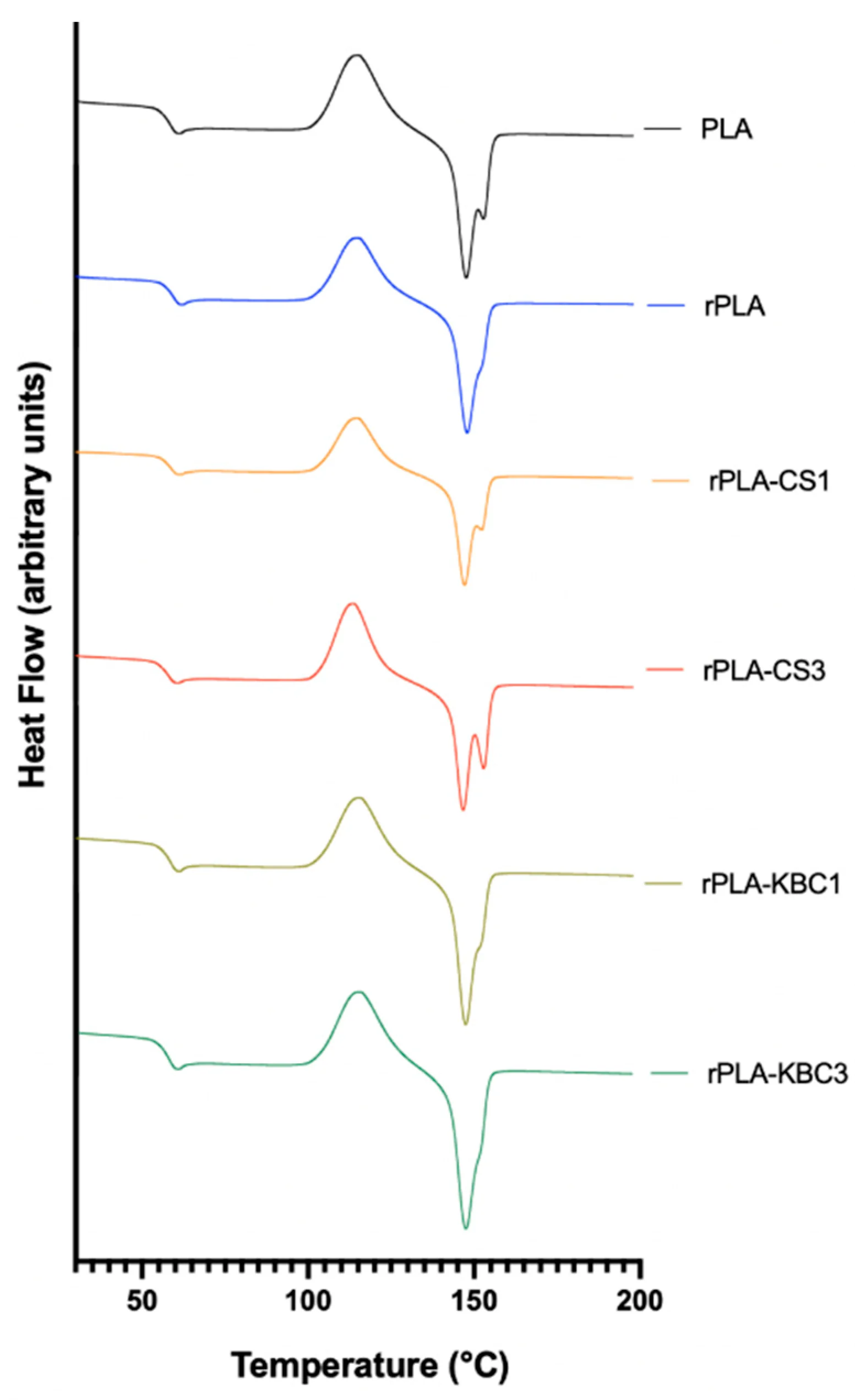
DSC thermograms of PLA-based films reinforced with cauliflower stem powder (CS) and kombucha bacterial cellulose (KBC).

Differential scanning calorimetry (DSC) was performed to evaluate the thermal processing behavior and crystallinity of the films. Representative thermograms are shown in Figure 3, while the thermal parameters obtained from the second heating scan are summarized in Table 4. All formulations exhibited similar glass transition (T_g_), cold crystallization (T_cc_), and melting (T_m_) temperatures, indicating that neither reprocessing nor filler incorporation substantially altered the characteristic thermal transitions of PLA under the processing conditions employed, in agreement with previous studies on mechanically reprocessed PLA [12]. These results are consistent with the ATR-FTIR analysis, which showed that the chemical structure of the polymer remained essentially unchanged after reprocessing and filler incorporation. Although the transition temperatures remained nearly constant, slight differences were observed in the cold crystallization and melting enthalpies, resulting in small variations in the calculated degree of crystallinity (X_c_). Reprocessed PLA (rPLA) exhibited a slightly higher crystallinity than neat PLA (2.7% and 2.3%, respectively), suggesting that the additional extrusion cycle did not impair the ability of the polymer chains to reorganize during heating. The incorporation of cauliflower stem powder (CS) slightly reduced the crystallinity of the films, particularly for rPLA-CS1, which exhibited the lowest X_c_ value (1.7%). Increasing the CS content to 3 wt.% resulted in a modest recovery of crystallinity (2.0%), although the values remained below those of rPLA. Since T_cc_ and T_m_ remained essentially unchanged, these differences appear to be associated with slight variations in the extent of crystalline organization rather than modifications of the crystallization mechanism. In contrast, the incorporation of kombucha bacterial cellulose (KBC) maintained crystallinity values comparable to those of neat PLA and rPLA, while rPLA-KBC3 exhibited the highest crystallinity among the evaluated formulations (3.2%). As shown in Figure 3, this formulation displayed a slightly more pronounced melting endotherm without appreciable changes in the transition temperatures, suggesting a modest increase in crystalline organization during heating. Overall, the DSC results indicate that reprocessing and CS or KBC filler incorporation had little influence on the characteristic thermal transitions temperatures of PLA, while only slight differences were observed in the degree of crystallinity. Since all formulations exhibited relatively low crystallinity values (1.7–3.2%), indicating that the films were predominantly amorphous, crystal development was limited and likely occurred predominantly during the DSC heating scan rather than during film processing. Similar low crystallinity values have been previously reported for mechanically recycled PLA-based films, whose crystalline organization is strongly dependent on the thermal history and processing conditions [17]. The low crystallinity obtained can be associated with the limited ability of rPLA chains to reorganize into ordered crystalline domains during the DSC heating scan used here. Moreover, considering the narrow range of X_c_ values observed among the film formulations, crystallinity is unlikely to be the main factor governing the small variations observed in the mechanical, morphological, and barrier properties discussed in the following sections.

### 3.5. Mechanical Properties

The tensile properties of the films are summarized in Table 5. Reprocessing caused slight changes in Young’s modulus (E), tensile strength (σ_t_), and elongation at break (ε_b_) compared with neat PLA. However, no statistically significant differences were observed between PLA and rPLA for any of the tensile test parameters (*p* > 0.05), indicating that the additional extrusion cycle did not substantially compromise the mechanical performance of PLA. This indicates that the decrease in molecular weight observed after reprocessing did not turn into a significant deterioration of the mechanical performance. Similar behavior has been reported for reprocessed PLA films, where early reprocessing cycles produced only minor changes in tensile properties without significantly affecting the structural integrity of the material [12,40]. The incorporation of bio-based fillers affected the tensile properties of the films, although the extent of this effect depended on the filler type and concentration. Considering the very low and similar crystallinity values obtained for all composite formulations (X_c_ = 1.7–3.2%, Table 4), the differences in mechanical behavior are unlikely to be primarily governed by changes in crystallinity. Instead, the mechanical response of these predominantly amorphous films is expected to be more strongly influenced by filler–matrix interactions.

In general, both CS and KBC reduced the elongation at break, indicating a more brittle mechanical response after filler incorporation. This behavior is commonly observed in PLA and rPLA-based biocomposites and can be ascribed to the restricted mobility of the polymer chains due to the incorporation of rigid lignocellulosic fillers [15]. This behavior is commonly reported for PLA-based biocomposites and is generally attributed to the restricted mobility of the polymer chains imposed by rigid cellulosic fillers, together with heterogeneous filler dispersion and limited interfacial adhesion, which reduce the efficiency of stress transfer across the polymer–filler interface [17,41].

Among the reinforced formulations, films containing CS exhibited the greatest deterioration in mechanical performance, particularly at 3 wt.%, where a marked and significant reduction in tensile strength were observed compared with neat PLA and rPLA (*p* < 0.05). This marked decrease can be attributed to the restriction of polymer chain mobility probably due to the more heterogeneous nature of CS than KBC. As it was already mentioned during TGA results discussion, CS is a complex lignocellulosic material containing mainly cellulose, hemicellulose and lignin, as well as other plant-derived components. Such heterogeneity in the composition of CS may result in less favorable interfacial interactions between CS and the hydrophobic PLA matrix, limiting effective interfacial bonding and promoting local stress concentration at the polymer–filler interface. In fact, this effect may become more pronounced at higher filler loadings, contributing to the marked decrease in tensile strength observed for rPLA-CS3.

For Young’s modulus, the effect was dependent on the filler loading levels. The rPLA-CS1 formulation showed intermediate values and due to the overlapping statistical groups, no significant differences were observed with respect to either PLA or rPLA. Nevertheless, despite the lack of statistical significance, this decrease represents an approximately 31% reduction in stiffness and it is relevant from a practical and engineering perspective, as it indicates a substantial loss of material stiffness that should be considered when selecting these formulations for packaging applications requiring mechanical rigidity, whereas rPLA-CS3 formulation exhibited the lowest Young’s modulus and differed significantly from neat PLA. These results suggest that increasing the CS content promoted structural discontinuities within the polymer matrix and/or lower polymer–filler interface interaction, reducing its ability to resist applied stress. In contrast, the incorporation of KBC resulted in a less pronounced reduction in mechanical properties. Although KBC-containing films also exhibited lower elongation at break than neat PLA and rPLA, their tensile strength remained higher than that of rPLA-CS3, particularly at the highest filler loading, while Young’s modulus was largely preserved, especially for rPLA-KBC3, which did not differ significantly from rPLA. The intermediate statistical grouping observed for rPLA-KBC1 also suggests that the reduction in stiffness was less pronounced than that observed for the corresponding CS-containing formulations. Similar observations have been reported for PLA composites reinforced with bacterial cellulose, where improved filler dispersion contributed to more efficient stress transfer across the polymer–filler interface [17,22]. These results indicate that the type of bio-based filler played a key role in determining the mechanical performance of the films. While both fillers increased the brittleness of the materials, KBC was more effective than CS in preserving the stiffness and tensile performance of the rPLA matrix, particularly at 3 wt.%, where the Young’s modulus of rPLA-KBC3 remained statistically comparable to that of rPLA. These findings highlight the potential of bacterial cellulose as a promising reinforcement for sustainable rPLA-based packaging materials. Despite some reduction in mechanical performance due to the incorporation of CSP and KBC, the developed composites provided antioxidant functionality with interest in active food packaging applications.

### 3.6. Field Emission Scanning Electron Microscopy (FESEM)

**Figure 4:**
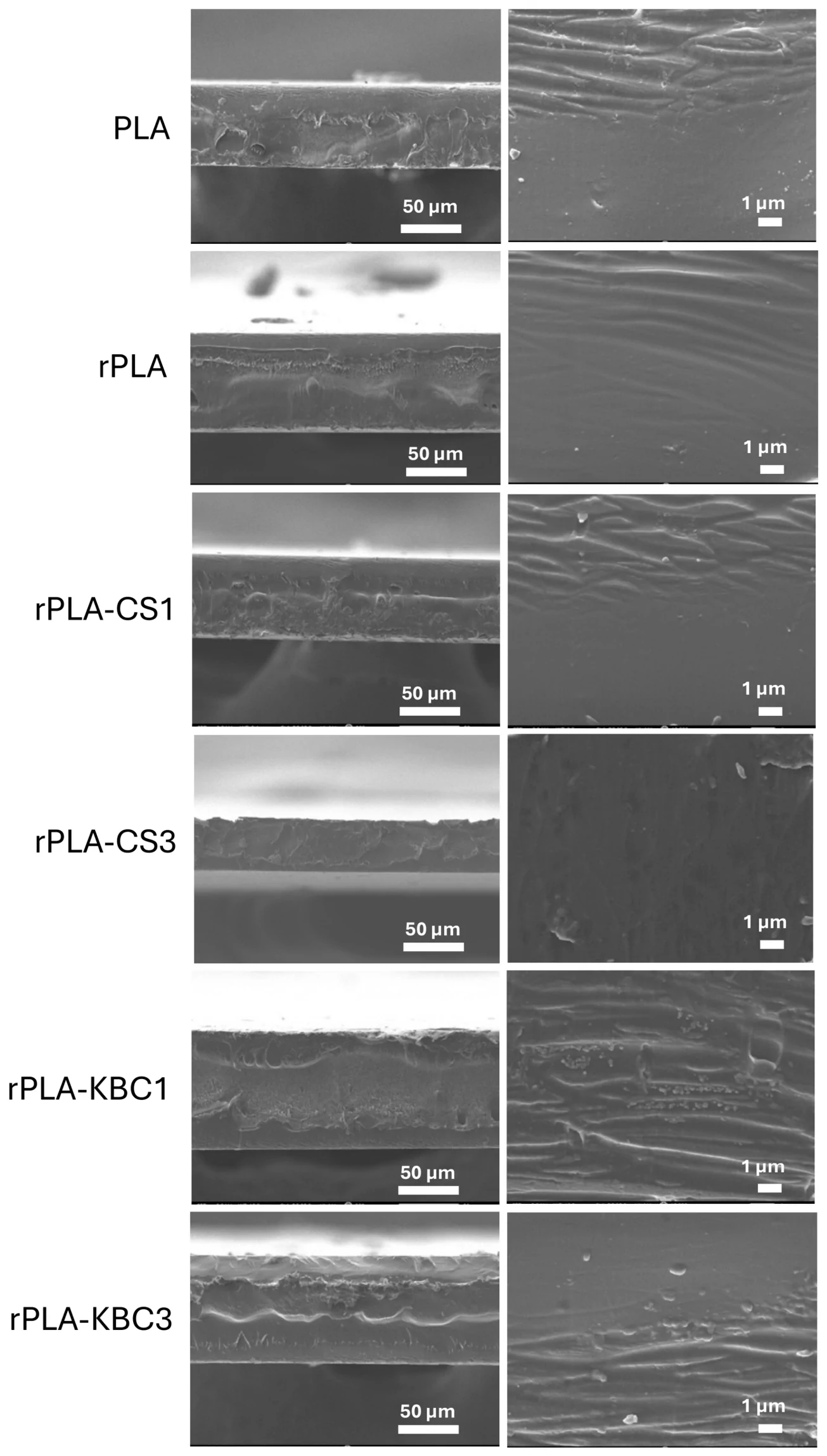
FESEM images of cryo-fractured cross-sections of PLA, rPLA and reinforced films containing cauliflower stem powder (CS) and kombucha bacterial cellulose (KBC) at 500× and 10,000× magnifications.

**Figure 5:**
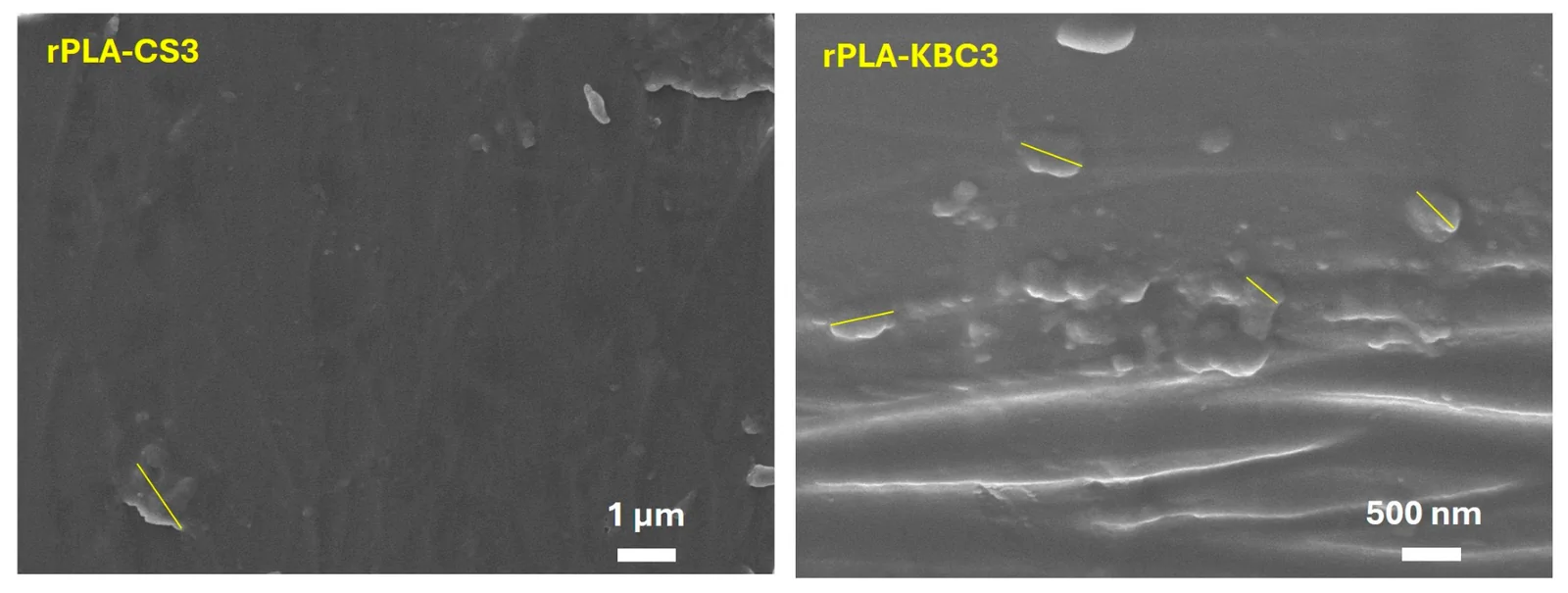
FESEM images of cryo-fractured cross-sections of rPLA-CS3 (performed at 10,000×) and rPLA-KBC3 (performed at 20,000×).

The cross-sectional morphology of the films was evaluated by FESEM at magnifications of 500× and 10,000× to assess the effect of reprocessing and filler incorporation on the structure of the rPLA matrix (Figure 4). Neat PLA exhibited a compact and continuous fracture surface with no evident structural defects, consistent with the typical brittle fracture morphology reported for PLA films [41]. After reprocessing, rPLA showed a very similar morphology, indicating that the additional extrusion cycle did not noticeably alter the bulk structure of the polymeric matrix in accordance with previous works [12,23]. These observations agree with the ATR-FTIR, thermal, and mechanical results, which showed only minor changes after reprocessing. The incorporation of CS and KBC produced only slight changes in the fractured morphology. At 500×, all formulations maintained continuous cross sections, although rPLA-CS3 exhibited a slightly rougher fracture surface than the other composites. At 10,000×, localized surface irregularities and small domains were observed in both CS- and KBC-containing films, but no evident interfacial voids, particle pull-out, or severe structural defects were detected. In Figure 5, small filler-related features could be observed embedded within the rPLA matrix, with larger features observed in rPLA-CS3 (1.3 µm), whereas in rPLA-KBC3 several submicrometric structures were detected (470 ± 75 nm). However, these observations do not allow a quantitative assessment of particle size or filler dispersion within the polymer matrix. Overall, the SEM observations suggest that the incorporation of bio-based fillers did not substantially modify the integrity of the rPLA polymeric matrix. The slightly rougher morphology observed for rPLA-CS3 is consistent with the poorer mechanical performance. Nevertheless, as at the scale evaluated by FESEM no evident interfacial voids or particle pull-out were detected in rPLA-CS3, the marked decrease in tensile strength cannot be directly explained by severe morphological defects. Meanwhile, the remaining formulations maintained continuous cross-sectional morphologies without evident defects. This behavior is in agreement with previous studies on PLA composites reinforced with kombucha-derived bacterial cellulose, where higher filler loadings resulted in a less homogeneous filler distribution [26].

### 3.7. Water Vapor Transmission Rate and Static Water Contact Angle

The interaction between packaging materials and moisture is a key factor affecting food preservation, as water vapor directly influences product stability and shelf life. Therefore, the water vapor transmission rate (WVTR) and static water contact angle (WCA) values of the developed films were determined and are presented in Table 6. Although the experimental design targeted a relative humidity (RH) of 90%, the chamber stabilized at approximately 70% RH during testing due to frequency of the measurements that requires to open the desiccator. Therefore, the WVTR values reported in this work should be considered as obtained under the actual experimental conditions (RH > 70%) rather than as standard values determined at RH > 90%. Nevertheless, since all formulations were simultaneously evaluated under the same WVTR experimental conditions, the obtained values allow a valid comparative assessment of the relative effect of CS and KBC incorporation on WVTR.

Neat PLA and reprocessed PLA (rPLA) exhibited similar WVTR values, indicating that the additional processing cycle did not significantly affect the water vapor barrier properties. This result agrees with the ATR-FTIR, SEM, and mechanical analyses, which showed that reprocessing caused only minor changes in the films. Similar observations were reported by Agüero et al. (2019), who found that a single reprocessing cycle did not significantly affect the morphology of PLA, whereas more noticeable changes were observed after subsequent reprocessing cycles [40]. Similarly, Sepúlveda-Carter et al. found that a single reprocessing cycle did not significantly affect the WCA or WVTR (tested at 21 °C RH above 70%) of PLA [12]. The incorporation of biobased fillers affected the barrier performance depending on filler type and loading levels. CS increased the WVTR, with rPLA-CS3 showing the highest value. Similar increases in water vapor transmission have been reported for PLA composites reinforced with cellulose microfibers, particularly at higher filler loadings, and have been attributed to the hydrophilic nature of cellulose together with the formation of preferential pathways for water vapor diffusion through the composite structure [42]. In contrast, rPLA-KBC1 maintained a barrier performance comparable to that of neat PLA and rPLA. A significant increase in WVTR was only observed for rPLA-KBC3, although it remained lower than that of rPLA-CS3. No significant differences were observed in the static water contact angle (WCA) among formulations, indicating that surface wettability remained essentially unchanged after filler incorporation. Therefore, the differences in WVTR are more likely related to the presence and concentration of the fillers rather than to changes in surface hydrophobicity or changes in crystallinity. Consistent with the SEM observations, only subtle morphological differences were detected among the films, suggesting that the more homogeneous morphology of the KBC-containing films and the better interaction of pure cellulose with rPLA matrix contributed to preserving the water vapor barrier properties of rPLA better than the CS-containing formulations, particularly at the lower filler loading.

### 3.8. Antioxidant Activity

The interaction between packaging materials and foodstuff is a key factor affecting food preservation, and it can be used as a positive property for the development of active packaging systems. The antioxidant activity of the developed films was evaluated by the DPPH radical scavenging assay using fatty food simulant D1 (Table 6). As expected, neat PLA and reprocessed PLA (rPLA) showed no detectable antioxidant activity, confirming that the polymer matrix itself does not contribute to radical scavenging under the tested conditions. In contrast, films containing CS and KBC exhibited measurable radical scavenging activity, with RSA values ranging from approximately 10% to 23%. Although a tendency toward higher RSA was observed with increasing filler content, particularly for rPLA-KBC3 which showed the highest mean RSA value, differences among composite formulations were not statistically significant (*p* > 0.05, Welch’s ANOVA). The relatively high standard deviations suggest that the variability of the particle intrinsic nature and composition may have masked differences between formulations. The antioxidant activity detected in the composite films is attributed to the incorporation of the biobased fillers, since neither PLA nor rPLA exhibited radical scavenging capacity. In the case of KBC, previous studies have reported that bacterial cellulose produced during kombucha fermentation can retain bioactive compounds from the fermentation medium, contributing to the antioxidant activity of the resulting materials [25]. While the mechanisms responsible for the observed activity were not investigated in this study, these results demonstrate that both CS and KBC provided antioxidant functionality to the rPLA films. Although KBC-containing formulations showed the highest RSA mean values, these differences were not statistically significant (*p* > 0.05) and should therefore be interpreted only as a trend. The antioxidant activity observed for KBC-containing films could be related to the ability of bacterial cellulose to retain bioactive compounds from the cauliflower infusion within its cellulose network during fermentation. This retention ability of bacterial cellulose structure may provide a protective effect against thermal degradation of these compounds during melt processing, allowing part of their antioxidant functionality to be preserved in the resulting films. Similar antioxidant activities have been reported for PLA active films containing green tea and rosemary extracts, where the incorporation of natural phenolic compounds conferred radical scavenging activity to the PLA matrix under comparable migration conditions in a fatty food simulant [43]. Nevertheless, it should be highlighted that although moderate, the antioxidant activity of the materials developed here showed higher RSA values than similar systems based on 3 times reprocessed PLA reinforced with kombucha cellulose fermented in yerba mate wastes [12]. Therefore, despite the somewhat reduction observed in the mechanical performance due to the incorporation of CSP and KBC, the developed composites provided antioxidant functionality with interest for active food packaging applications.

### 3.9. Disintegration Under Composting Conditions

**Figure 6:**
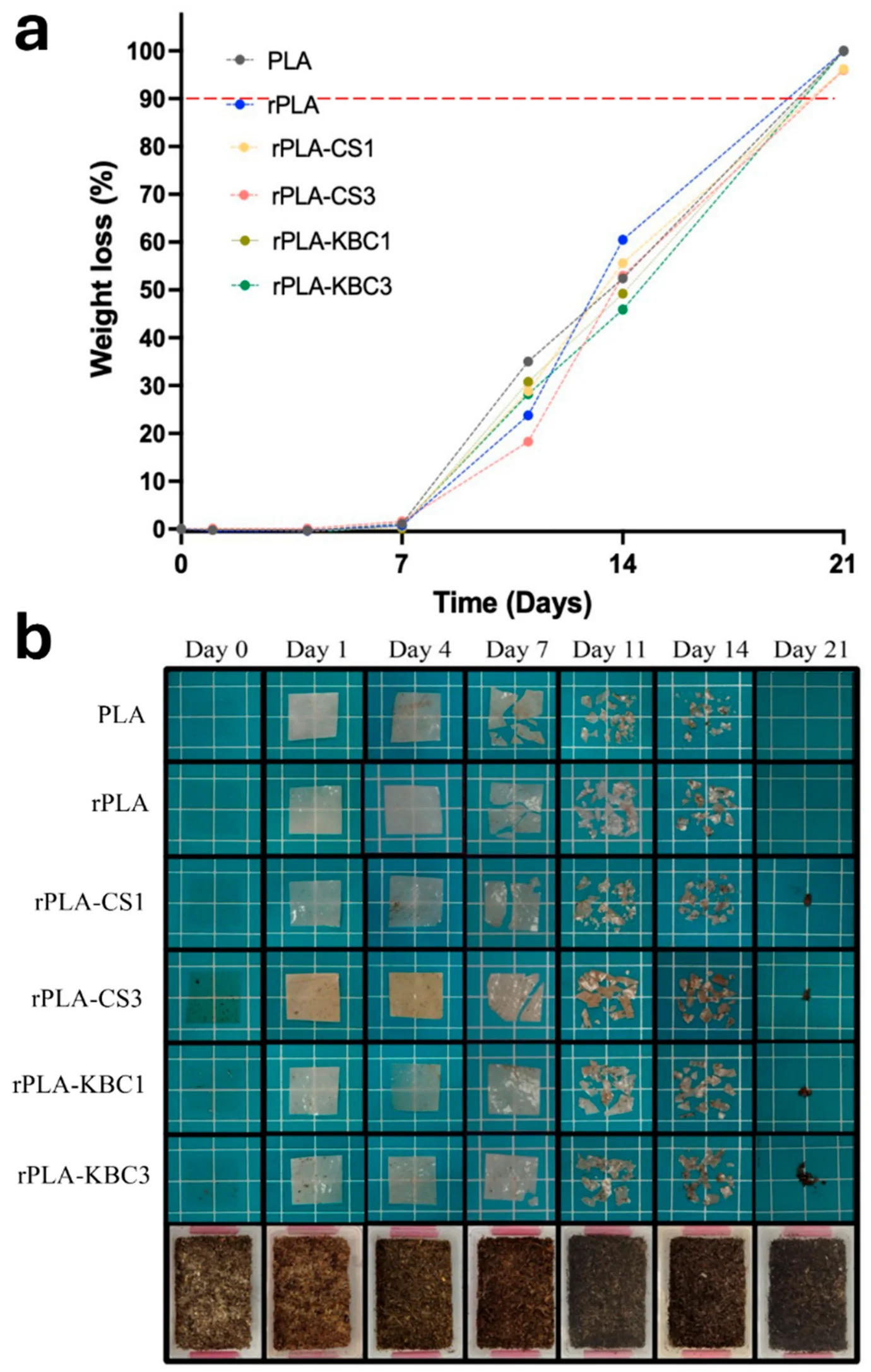
(**a**) Disintegration degree of PLA-based films reinforced with cauliflower stem powder (CS) and kombucha bacterial cellulose (KBC) under composting conditions as a function of time. The red dotted line indicates 90% disintegration. (**b**) Visual appearance of the films during the disintegration process at different incubation times under composting conditions. Results corresponding to neat PLA and rPLA films were adapted from [12] and reproduced here for comparison under the terms of the Creative Commons Attribution (CC BY 4.0) license.

Disintegration under composting conditions was evaluated to assess compostability as the end-of-life option for the developed rPLA-based films. The disintegration profiles are presented in Figure 6a, while the visual evolution of the films during composting test is shown in Figure 6b. According to ISO 20200:2024 and the compostability criteria commonly adopted in the literature, plastic materials are considered disintegrable when reaching at least 90% mass loss under controlled composting conditions [21,44]. In this study, all formulations reached complete disintegration within four weeks, confirming their disintegrable character under compost conditions. As previously reported, neat PLA and rPLA exhibited similar disintegration profiles, although rPLA showed a slightly faster weight loss during the intermediate stage of the assay [12,14]. The incorporation of bio-based fillers slightly affected the degradation rate depending on the filler type and loading. rPLA-KBC3 exhibited the slowest initial disintegration, whereas rPLA-CS3 showed a slightly faster degradation after the first week. Similar trends have been reported for PLA-based composites reinforced with cellulosic fillers, where cellulose can promote the disintegration process, while the presence of a second biodegradable polyester may slightly delay the degradation rate without compromising the overall compostability of the material [44]. However, these differences gradually diminished, and all formulations reached comparable disintegration levels by the end of the test. The visual observations (Figure 6b) were consistent with the quantitative results, showing progressive fragmentation of all films throughout the composting period. After 21 days, all formulations had almost completely disintegrated with only small fragments remaining, whereas compost color change (Figure 6b) because of the aerobic fermentation that finally resulted in dark humus soil. These results demonstrate that the incorporation of CS and KBC did not compromise the compostability of rPLA films, while providing additional functionality without affecting their end-of-life performance.

### 3.10. Limitations and Future Research Directions

Although the results obtained in this work show the potential of cauliflower stem-derived CS as a functional filler and as a substrate for active KBC production for incorporation into reprocessed PLA films, some aspects still need to be addressed before their application in real food-packaging systems. In particular, scale-up and reproducibility should be considered, since the composition of food-derived waste streams may vary depending on their origin, seasonality, and previous treatments and/or processing.

Future studies should evaluate CS recovery, KBC yield and productivity, carbon utilization efficiency, and resource requirements at larger scales to assess not only the technical feasibility, but also the environmental as well as the economic viability of the proposed approach.

Further formulation optimization should focus on preserving the functional benefits provided by CS and KBC while minimizing the reduction in mechanical performance. The use of plasticizers or compatibilizers, as well as a more comprehensive rheological and filler–matrix interfacial characterization, could contribute to the material’s final performance optimization. Finally, validation under realistic food-packaging conditions should include water absorption and solubility, migration and food-contact safety, stability of the antioxidant functionality during storage, and shelf-life studies using real food systems. Life cycle assessment (LCA) will be essential to determine the environmental impact of the final materials.

## 4. Conclusions

Kombucha bacterial cellulose (KBC) was successfully produced from cauliflower stem powder (CS), an underutilized agro-industrial by-product, and further incorporated, into a reprocessed PLA (rPLA) matrix to produce biobased composite films by melt extrusion followed by compression molding.

Reprocessing did not significantly affect the physicochemical and thermal transitions, structural properties, mechanical performance, water vapor barrier properties, or compostability of PLA, demonstrating the feasibility of reintroducing processed PLA into new material cycles. Meanwhile, the incorporation of biobased fillers influenced the film properties depending on the filler type and loading. Although both particles reduced the thermal stability, this reduction was generally less pronounced KBC-based composites than for CS-based counterparts, but it should be highlighted that all composites were thermally stable at the PLA processing temperature and the intended use. Meanwhile, both fillers reduced the mechanical performance of the films, with KBC showing a lower negative impact than CS, leading to a good mechanical performance balance without significant differences among KBC loading levels. Regarding functional properties, KBC better preserved the water vapor barrier performance at low filler loadings, whereas both fillers provided measurable antioxidant activity despite the absence of significant differences among formulations. Furthermore, all films achieved complete disintegration under lab-scale composting conditions within 21 days, confirming that filler incorporation did not compromise their compostability. Thus, this study demonstrates the potential of combining reprocessed PLA with biobased fillers obtained from cauliflower stems to develop biobased and recycled materials with enhanced functionality and with potential composting as end-of-life option. These findings contribute to circular economy strategies by simultaneously valorizing agro-industrial and HoReCa residues and extending the service life of PLA through mechanical recycling, offering a promising approach for future active food packaging applications.

## Figures and Tables

**Table 1:** Composition and formulation codes of PLA-based composite films reinforced with cauliflower stem powder (CS) and kombucha bacterial cellulose (KBC).

Sample Code	PLA [wt.%]	CS [wt.%]	KBC [wt.%]
PLA	100	-	-
rPLA	100	-	-
rPLA-CS1	99	1	-
rPLA-CS3	97	3	-
rPLA-KBC1	99	-	1
rPLA-KBC3	97	-	3

**Table 2:** Intrinsic viscosity and viscosity-average molecular weight of the PLA and rPLA pellets and films.

Sample	[η] [mL/g]	M_v_ [g/mol]
PLA pellet	105.25 ± 1.34	113,776 ± 1915
rPLA pellet	98.96 ± 2.13	104,903 ± 2975
PLA	97.38 ± 3.33	102,703 ± 4624
rPLA	93.37 ± 2.74	97,166 ± 3760

**Table 3:** Summary of thermal parameters obtained by TGA for rPLA-based films reinforced with cauliflower stem powder (CS) and kombucha bacterial cellulose (KBC).

Sample	T_5%_ [°C]	T_max_ [°C]	Mass Residual [%]
PLA	323.5	364.0	1.0
rPLA	325.3	365.9	1.7
rPLA-CS1	312.8	352.7	3.3
rPLA-CS3	304.3	344.5	3.4
rPLA-KBC1	312.6	352.4	1.5
rPLA-KBC3	311.9	349.2	3.2

**Table 4:** Summary of DSC thermal parameters of PLA-based films reinforced with cauliflower stem powder (CS) and kombucha bacterial cellulose (KBC).

Sample	T_g_ [°C]	T_cc_ [°C]	ΔH_cc_ [J/g]	T_m_ [°C]	ΔH_m_ [J/g]	X_c_
PLA	59.0	116.3	25.4	149.1	27.6	2.3
rPLA	59.9	116.1	26.9	149.4	29.5	2.7
rPLA-CS1	59.2	116.2	21.6	148.8	23.2	1.7
rPLA-CS3	58.2	114.6	27.3	148.3	29.0	2.0
rPLA-KBC1	59.1	116.4	24.5	149.1	26.6	2.3
rPLA-KBC3	58.5	116.8	25.6	149.2	28.5	3.2

**Table 5:** Summary of tensile properties of PLA-based films reinforced with cauliflower stem powder (CS) and kombucha bacterial cellulose (KBC).

Sample	E [MPa]	σ_t_ [MPa]	ε_b_ [%]
PLA	3363 ± 304 ^a^	62 ± 7 ^a^	5.0 ± 1.1 ^a^
rPLA	2904 ± 425 ^ab^	59 ± 17 ^a^	4.8 ± 1.3 ^a^
rPLA-CS1	2313 ± 512 ^bc^	47 ± 6 ^ab^	2.7 ± 0.9 ^b^
rPLA-CS3	1521 ± 326 ^c^	18 ± 5 ^c^	1.9 ± 0.3 ^b^
rPLA-KBC1	2357 ± 532 ^bc^	47 ± 17 ^ab^	2.5 ± 0.6 ^b^
rPLA-KBC3	2877 ± 384 ^ab^	41 ± 14 ^b^	2.2 ± 0.4 ^b^

E: tensile modulus; σ_t_: tensile strength; ε_b_: elongation at break. Values are expressed as mean ± standard deviation (*n* = 5). Different lowercase letters within the same column indicate significant differences among samples (*p* < 0.05), according to one-way ANOVA followed by Tukey’s multiple comparison test.

**Table 6:** Surface, barrier and functional properties of PLA-based films reinforced with cauliflower stem powder (CS) and kombucha bacterial cellulose (KBC).

Sample	WVTR [g·m^−2^·day^−1^]	WCA [°]	RSA [%]
**PLA**	30.5 ± 1.4 ^a^	76.1 ± 2.5 ^a^	-
**rPLA**	31.0 ± 2.9 ^a^	76.5 ± 2.1 ^a^	-
**rPLA-CS1**	37.8 ± 8.6 ^b^	76.2 ± 2.9 ^a^	11.8 ± 3.6 ^a^
**rPLA-CS3**	73.1 ± 3.8 ^c^	75.4 ± 2.8 ^a^	10.8 ± 2.3 ^a^
**rPLA-KBC1**	27.6 ± 3.0 ^a^	75.5 ± 3.0 ^a^	14.8 ± 5.1 ^a^
**rPLA-KBC3**	52.6 ± 8.9 ^b^	75.6 ± 2.8 ^a^	22.6 ± 9.3 ^a^

WVTR: water vapor transmission rate. RSA: radical scavenging activity, determined using DPPH method in food simulant D1. WCA: static water contact angle. Different lowercase letters in the same column indicate significant differences among samples (*p* < 0.05), according to one-way ANOVA followed by Tukey’s multiple comparison test. No significant differences were observed for RSA (%) (Welch’s ANOVA, *p* > 0.05; Dunnett’s T3 post hoc test).

## Data Availability

The data presented in this study are openly available in the e-cienciaDatos repository (Consorcio Madroño) at https://doi.org/10.21950/QRAJA4.

## Abbreviations

The following abbreviations are used in this manuscript:

PLA	Poly(lactic acid)
rPLA	Reprocessed poly(lactic acid)
CS	Cauliflower stem
KBC	Kombucha bacterial cellulose
FTIR	Fourier transform infrared spectroscopy
TGA	Thermogravimetric analysis
DSC	Differential scanning calorimetry
SEM	Scanning electron microscopy
WVTR	Water vapor transmission rate
WCA	Water contact angle
RSA	Radical scavenging activity
DPPH	2,2-Diphenyl-1-picrylhydrazyl
